# A Society of General Internal Medicine Position Statement on the Internists’ Role in Social Determinants of Health

**DOI:** 10.1007/s11606-020-05934-8

**Published:** 2020-06-09

**Authors:** Elena Byhoff, Shreya Kangovi, Seth A. Berkowitz, Matthew DeCamp, Elizabeth Dzeng, Mark Earnest, Cristina M. Gonzalez, Sarah Hartigan, Reena Karani, Milad Memari, Brita Roy, Mark D. Schwartz, Anna Volerman, Karen DeSalvo, On behalf of the Society of General Internal Medicine

**Affiliations:** 1 Department of Medicine, Institute for Clinical Research and Health Policy Studies Tufts Medical Center, Tufts University School of Medicine, Boston, MA USA; 2 Department of Medicine, University of Pennsylvania, Philadelphia, PA USA; 3 Department of Medicine, University of North Carolina, Chapel Hill, NC USA; 4 Department of Medicine, University of Colorado, Aurora, CO USA; 5 Department of Medicine, University of California, San Francisco, CA USA; 6 Department of Medicine, Albert Einstein College of Medicine, Montefiore Medical Center, Bronx, NY USA; 7 Department of Medicine, Virginia Commonwealth University, Midlothian, VA USA; 8 Department of Medicine, Medicine and Geriatrics and Palliative Medicine Icahn School of Medicine at Mount Sinai, New York, NY USA; 9 Departments of Medical Education, Johns Hopkins University School of Medicine, Baltimore, MD USA; 10 Department of Medicine, Yale Medicine, New Haven, CT USA; 11 Departments of Population Health and of Medicine, NYU Grossman School of Medicine, New York, NY USA; 12 Department of Medicine, University of Chicago, Chicago, IL USA; 13 Google, Mountain View, CA USA

For if medicine is really to accomplish its great task, it must intervene in political and social life. It must point out the hindrances that impede the normal social functioning of vital processes, and effect their removal.

-Rudolf Virchow, 1849.

The Society of General Internal Medicine (SGIM) represents the world’s leading academic general internists, dedicated to creating a just system of care in which all people can achieve optimal health. SGIM recognizes that to achieve this vision, we must expand our reach beyond the medical office and hospital bedside to identify and address the broader structures and living conditions that influence health—the social determinants of health (SDOH). Centuries of institutionalized oppression in the form of racism, sexism, and other forms of bigotry have created and perpetuated disadvantage. These underlying social values have translated into public policies and structures which affect the distribution of money and power across society. These in turn have shaped living conditions and access to resources, which influence health behaviors and access to care, and ultimately health outcomes.^1, 2^ SGIM acknowledges the full spectrum of SDOH including upstream policies, midstream environmental and behavioral factors, and downstream individual and clinical issues.^3–5^ We highlight an important distinction between societal SDOH that require policy and systems-level change, with downstream unmet individual social needs, like homelessness or food insecurity. The entire range of SDOH impacts the work we do, our ability to care for our individual patients, our partnerships with local community organizations, and our impact on population health and equity. SDOH should also be integrated in how we teach future physicians and collaborate with our colleagues in public health, social work, government, research, and partners in non-health sectors.

In this position statement, we draw from medicine, public health, sociology, and ethics to contextualize the daily relevance of upstream SDOH and downstream social risks for SGIM members. We support statements issued by the American College of Physicians, the American Academy of Pediatrics, the National Academy of Medicine, and others.^6–8^ We build upon these to offer a set of positions specifically tailored for the academic general internist. SGIM members are practicing physicians, health system leaders, educators, researchers, and advocates. We articulate strategies for how each of these roles can be leveraged to address SDOH and social needs, and ultimately to improve health and health equity.

## A CASE STUDY

A 67-year-old woman with hypertension presents, complaining of dizziness. She was in the office a month earlier with the same complaint. At that visit, her blood pressure was 118/62, a bit lower than usual. She stopped one of her blood pressure medications as instructed, but her symptoms persisted. The dizziness interferes with her work as a store cashier. An EKG and complete physical exam fail to explain her dizziness. You ask her to share more about her symptoms. She tells you her daughter and grandchildren recently moved back in with her after their rent was raised. Money is tight. The local food pantry is only open one day a week, and often the line is so long that she cannot afford to wait and miss work. She has cut back on meals. Her landlord has discovered that she has new family members living in the unit who are not on the lease and is threatening eviction.

Her physician is witness to the impact of upstream SDOH on her clinical presentation. Generations of unjust policies created these downstream effects: 50 years ago, she was unable to buy a home in desirable neighborhoods as a result of federal redlining policies restricting “negroes or foreigners” from buying homes in A-rated neighborhoods.^9^ Her current community—its school systems, food stores, and transit hubs—suffered decades of disinvestment. Her financial challenges result from a scarcity of well-paying jobs, cliff effects for benefits like the supplementary nutrition assistance programs and Section 8 housing, and regressive economic policies that tie social security to lifetime earnings. The cumulative effects of these stressors she’s experiencing, combined with structural and institutional gender and racial bias, contribute to her increased risk of poor health outcomes.

While the evidence is clear that these SDOH and social needs directly impact health, the doctor’s role has been less well-defined. The time has come to define our role. As illustrated in this vignette, poor health and health inequity are the consequence of multiple complex and intersecting problems. Thus, we propose countervailing actions across our spheres of influence as physicians, health system leaders, educators, researchers, and advocates.

## EXECUTIVE SUMMARY: ACTION ACROSS OUR SPHERES OF INFLUENCE

SGIM as an organization and its members should and will commit to the following positions:

- As practicing *physicians*, we should learn about our patients as people through relationship-centered communication and self-reflection about how our own biases might interfere with our ability to deliver equitable care. We should hire and work in interprofessional care teams to ensure we can provide whole-person care to our patients. Interprofessional teams should include social care specialists such as community health workers or peer navigators to integrate medical and social aspects for whole-person care, as well as the expertise of nurses, pharmacists, mental health providers, and others.
- As *health system leaders*, we should encourage our organizations to partner with community members and community-based organizations. Our health systems should also leverage their own economic and political footprints as anchor institutions that source locally, pay a living wage, and foster trust with and invest in local communities. Leadership should prioritize workplace diversity and develop family-friendly workplace policies.
- As *educators*, we should include social and relational competency as a necessary qualification for future physicians. We support a holistic medical education process that relies on multi-modal assessments. We should develop curricula with SDOH learning objectives for every stage of physician education and evaluation. Undergraduate and graduate medical education should include experiential curricula for SDOH to not only make future physicians aware of how social and environmental circumstances outside the hospital are critical to health but also how to effectively advocate for improvement. We should ensure that accreditation and licensure examinations assess communication, cultural humility, bias and stereotyping behaviors, and structural competency. Medical and continuing education curricula should teach how structures—the large-scale organization of social, economic, and political power—impact health,^10^ with the goal of improving patient outcomes and care delivery, rather than learner test scores.
- As *researchers*, we should use science as a tool of inclusion by encouraging authentic partnerships with community members at all levels—involving patients and families in the design of social needs interventions and the prioritization of research questions, including community members in decision-making committees, and collaborating with community-based organizations in implementation. We should partner with researchers in other fields to identify interdisciplinary solutions to complex social problems that result in poor health. We should demand rigor in the evaluation of social interventions and policies to ensure that the best work moves forward.
- As *advocates and as a professional society*, we should advocate for the assessment of health impacts of key federal policies. We should advocate alongside public health and community partners to ensure the execution of the Affordable Care Act’s Community Health Needs Assessments better align across the communities and neighborhoods we serve.^11, 12^ Finally, we should advocate to the federal and state governments to create financial structures that share dollars from all payer- and incentive-driven savings programs from healthcare and into other public sectors such as housing.

Across these spheres, science and ethics will be guiding principles. The scientific formation and testing of new ideas, interventions, and policies will be critical if we are to achieve real impact. The ethical principles of autonomy, beneficence, non-maleficence, and justice are all equally critical. Given history and existing power dynamics, the best intentions do not inoculate us from unintentional consequences.

## ACTION ACROSS OUR SPHERES OF INFLUENCE

### *SGIM members, first and foremost, are physicians and practice leaders. In these roles, we should create teams who support patients as people.*

#### Engage in relationship-centered communication during patient visits

Primary care doctors and hospitalists should have holistic and strengths-based conversations with patients about social needs.^13, 14^ Efforts to increase these kinds of discussions cannot be limited to pro-forma screening based solely on incentives to meet quality metrics or reimbursement bonuses. Relationship-centered communication includes empathetic conversations, shared decision-making, and appreciative inquiry, which includes asking patients about their “life stories” including childhood experiences, life milestones, and key relationships. This approach allows patients to feel “seen” as people, rather than as a list of problems or diagnoses.^15, 16^ Including open-ended questions does not add length to the patient encounter^17^ and lays a foundation for a therapeutic alliance which can make clinical decision-making more efficient and effective. Employing a person-centric approach increases patient satisfaction and reduces physician burnout.^17^ Physicians must respect patient autonomy in approaching these conversations, explicitly identifying patient priorities and desire for healthcare system involvement in their social needs. Physicians should also engage in efforts to enhance self-awareness, including an examination of how their own background and life experiences influence their attitudes towards and interactions with patients from socially disadvantaged groups.

#### Embrace interprofessional team-based care

Identifying and addressing complex medical and social needs in a clinical setting requires a multidisciplinary care team. These teams should include members from across the healthcare and social care professions, including social workers, community health or peer navigators, nurses, pharmacists, mental health, and front-line or administrative support staff. Movement towards team-based approaches, where physicians can rely on colleagues with expertise in community-based and other social services, will reduce physician burden and increase the capacity of the care team to identify meaningful solutions for social needs. Building such team-based expertise can ensure that we provide tailored support addressing a range of our patients’ social and behavioral needs to achieve health.^18^

### SGIM members are physician administrators and health system leaders. In these roles, we should partner with and support local communities.

#### Buy instead of build community-based social services

While addressing social needs is somewhat new to healthcare, it is not new to community-based organizations that have long been providing needed services and supports. Health systems should avoid building de novo social care programs that can create unnecessary duplication of existing social services, often at a higher cost with less community input. We should reach out to existing community members and organizations for their expertise and partnership in social service delivery and community outreach. Dollars that should be supporting local communities, through such mechanisms as Community Needs Assessments and Community Benefits, often remain within the healthcare organizations, minimizing impact on community well-being.^19–21^ We should ensure that our health systems partner with community members and organizations to ensure the flow of dollars to support existing services, such as after school programs, food banks, and homeless shelters. Physician leaders can be catalysts for community partnership, opening dialogue into how healthcare systems can listen to community members, and provide services and investments identified and prioritized by the communities they serve. As organizational leaders, these physicians can also establish and monitor metrics that measure progress toward agreed on areas of focus.

#### Leverage economic and political power to support communities

Healthcare organizations can intervene on upstream SDOH simply by redirecting how they spend their money and influence. “Anchor institutions” hire individuals from underserved communities, prioritize local and minority-owned vendors, create local financial investment strategies, hire a diverse workforce, institute family friendly policies, and pay employees a living wage.^22^ The National Academy of Medicine’s anchor institution approach to addressing SDOH has been endorsed by a growing collaborative of healthcare delivery organizations and can help health systems to take action.^23, 24^

#### Develop and nurture trust-based relationships with community institutions targeting health and health equity

Healthcare organizations should make institutional commitments to respectful practices for community engagement.^25^ Organizational leaders can and should establish systems and monitor adherence to these practices among all of the health professionals, faculty, learners, and administrators.

### SGIM members are educators who train future physicians across all medical specialties. In these roles, we should integrate multi-modal SDOH curricula and assessments throughout physician training and licensure.

#### Prioritize humanism and empathy in medical school admissions

The medical school admissions process overemphasizes didactic achievement compared to interpersonal skills and emotional intelligence. We advocate for a holistic approach to medial school admissions to ensure that the pipeline of future doctors includes a diverse pool of candidates enriched with traits such as empathy, humility, and self-awareness. Increasing diversity in students and trainees will have significant positive long-term impacts on the culture of medicine, breaking down traditional doctor-patient hierarchies and improving patient care.^26–28^

#### Prioritize and institutionalize SDOH curricula in all aspects of medical education, including continuing medical education

We support critical service learning experiences as part of medical education curricula. Medical training must not only fulfill the classic core competencies (e.g., physiology, biostatistics) but also encompass structural competence, communication, relationship-centeredness, and cultural humility. These constructs are challenging to teach in a didactic format, which is why medical schools sometimes offer experiential “service learning,” a pedagogical method in which students work in communities in order to expand their knowledge. Service-learning—which often takes shape through projects such as free student-run clinics or health education fairs—can perpetuate health inequities and reinforce implicit biases if not thoughtfully designed. We should ensure that medical school and medicine residency curricula are informed by the notion of “critical service learning” wherein community members work alongside with students, rather than simply receiving services.^10, 29, 30^ Critical service-learning emphasizes dialogue with community members on the underlying causes of disparities. This dialogue is intended to build structural competency, which is an ability to understand illness as a downstream result of structural injustices and SDOH. Examples of critical service learning rotations include experiential training with community-based organizations that address SDOH or a community health worker–led medical school rotation.^31^ For practicing physicians, CME should include SDOH competencies to ensure all physicians are aware of the scope of social and political impacts on SDOH for patients, how best to include social needs into routine patient care, and highlight provider implicit biases that perpetuate health inequalities.^32^

#### Revisit outcomes of interest for SDOH education and training

Moving away from strict didactic learning to more multi-modal or experiential learning requires innovative assessments. Accreditation and licensure bodies across the continuum of medical education (i.e., LCME, ACGME, and ACCME) should shift focus to patient outcomes as learners understand and incorporate SDOH in their clinical practice. In addition to including SDOH in UME, GME, and CME curricula, SDOH should be included in clinical skills assessments with a focus on impact in patient perceptions of care. We advocate for these changes in the USMLE as well and call upon the NBME to develop and implement meaningful assessments of SDOH within existing exams.

### SGIM members are scientists, grant reviewers, and leaders within research funding agencies. In these research roles, we should generate and promote interdisciplinary and community-engaged science. We should identify and use grading systems for social interventions to minimize evidence-to-practice gaps.

#### Using rigorous scientific methods, built on the existing evidence to identify and test SDOH interventions

Researchers in medicine, nursing, public health, sociology, and economics have reached consensus that poverty impacts health across the life course. Currently, many SDOH “solutions” are being developed without evidence-based hypotheses or using scientific principles to identify and evaluate them. SDOH interventions and policies should be constructed with care, and build upon social and behavioral scientific disciplines now confronting structural inequality; social epidemiology,^33^ psychology,^34^ education,^35^ and economics,^36^ are replete with relevant theory and empirical evidence that should inform the development of new SDOH interventions.^37^

#### Revise research funding priorities to include interdisciplinary and community-focused research

Many researchers also serve as reviewers for federal and philanthropic grants. Most federal and philanthropic research funding focuses on disease-specific interventions or outcomes. This kind of research is designed to treat patients and not communities. As research reviewers, we can influence funding priorities and in so doing increase the workforce diversity of physician researchers by prioritizing innovative work focused on community health and health disparities.^38^ Increasing the diversity of researchers in the field, and increasing funding opportunities for community-based research approaches, will increase the speed at which interventions are identified and tested and allow for new innovation from a previously underfunded group of researchers. Career development awards are particularly important to foster a generation of researchers with a deep understanding and commitment to reducing health disparities through community-engaged methods.

#### Science should be used as a tool of inclusion

Specific research methods that include community priorities and feedback are critical to ensure interventions and approaches to SDOH align with the communities and patients for whom they are designed. We advocate for approaches such as Participatory Action Research and Community-Based Participatory Research,^39–41^ which are designed to ensure that patients and families with lived experience are included at all stages of research including design, execution, participation, and dissemination. Outcomes of interest for our patients and families should be prioritized.

#### Identify and implement an evidence grading system

Currently, there are large evidence-to-practice gaps in the uptake of social interventions and policies. Many evidence-based effective interventions—such as nurse home visits for pregnant women, tailored support from community health workers, or housing coupled with intensive care management—remain underutilized.^42–46^ We support increased use of implementation science methods to increase the uptake and effectiveness of evidence-based practices for social interventions. We should use and build upon evidence grading systems such as the USPSTF, or Community Guide; these will ensure that investments have the greatest impact while highlighting knowledge gaps that can benefit from continued research. When causal inference is required, newer methods of randomization—pragmatic, adaptive, cross-over, and stepped wedge trials—which are used widely in global and public health, can help to ensure unbiased evaluation of social and community-based interventions.^47–55^ Because health-related social needs interventions are often complex and may be context-dependent, research should include mixed method designs that allow us to better understand why interventions have the results they do, and for whom, using qualitative methods.

### SGIM and its members are influential in evaluating and advocating for health-related policies. We should formally assess the health impacts of key policies and advocate for regulations that redirect resources from healthcare to other public sectors.

As general internists, we are in a unique position to identify patterns that lead to poor health outcomes. Physicians should identify and call out the upstream policy and structural factors that impact our patients and the populations we serve and advocate for policy and structural changes. Adverse SDOH are a consequence of long-standing policies, cultures, and institutions derived from our nation’s history of racism and exclusion. Therefore, direct policy action will have the most far-reaching impact on improving health, equity, and well-being. In our role as advocates, with more political capital than many other professionals, policy considerations should also align with our ethical and research-driven standards.

#### SGIM advocates for a “health in all policies” approach for federal, state, and local public and private sector policy

SGIM engages in legislative and advocacy priorities in line with our mission to create a just system of care in which all people can achieve optimal health. As a first step towards ensuring health in all policies, we propose the development of health impact assessments in policy-making. Health impact assessments will better ensure understanding of the intended and unintended health impacts of key federal policies. Similar to how the Congressional Budget Office (CBO) scores federal policy on its projected fiscal impact, we as physicians, who see and manage the downstream health consequences of many policies, advocate for a comparable health impact score. CBO or another federal agency can score proposed bills by estimating the population change in health-adjusted life expectancy.^56, 57^ Healthcare comprises 18% of the GDP.^58^ Implementation of policies that worsen health, resulting in more healthcare spending and utilization that could otherwise be avoided, has significant ripple effects. SGIM should advocate with the CBO and the Centers for Medicare and Medicaid Services (CMS) to develop health impact assessment methodology and partner with experts in academic medicine, public health, epidemiology, and economics, to understand the intended and unintended health consequences of any new federal policy. Policy-makers should also first do no harm.

#### Services and supports for SDOH require appropriate funding and reimbursement

Currently, systems for financing SDOH are siloed and insufficient. Fee-for-service-based funding strategies (paying for volume rather than value) need to be revised to incentivize health systems to invest in strategies to identify and engage with adverse SDOH facing the communities they serve. This should include increased focus and enforcement for community benefit plans by hospitals, an IRS requirement to maintain their tax-exempt status. Funding models should include sustainable and flexible reimbursement models to incentivize the use of interdisciplinary care teams, and to expand the impact of health systems by linking them with community-based resources. These changes require sufficiently resourcing primary care to build interprofessional, multidisciplinary teams with sufficient capacity and bandwidth to integrate health and social care.^59^

#### Public and private payers should develop payment methodologies that avoid the medicalization of SDOH

Medicalization occurs when non-medical issues become defined and treated as medical problems. SDOH are structural and environmental circumstances that lead to downstream social risks that have direct health consequences. By incentivizing healthcare organizations and payers to engage in addressing SDOH, real dangers exist. If a private insurance company subsidizes housing, does this mean that our patient’s home is now tied to their health plan? If their insurer denies certain medications or services, but they provide housing, how can an individual make a fair decision about medical care if it comes at the expense of losing their home? If insurers and healthcare organizations are building housing, or opening food banks, they are now positioned to limit access to only their patients, or only specific patient groups that demonstrate high enough costs. This raises profound ethical concerns.

#### We advocate for policies to encourage large-scale investment in social services sectors

SDOH are intricately linked to poverty. We advocate for anti-poverty policies at the local and federal level, including investment in housing and income supports. As specific social policies, these are evidence-based areas of investment to reduce the burden of poverty, decrease stress, and improve health outcomes for all. This is likely to require tough choices, redirecting some public funding from healthcare to these other sectors.

## SUMMARY

SGIM recognizes the fundamental importance of social circumstances in health.^60^ As advances in the biomedical model have led to significant progress in the fight against disease, our commitment to understanding and addressing social drivers of health like those faced by our patient in the case study must be renewed. Physicians as advocates can influence change along a spectrum, from the individual patient encounter, teaching learners, our clinical practices, our organizational priorities, our research agendas and discoveries, our communities, and policy. SGIM acknowledges the importance of SDOH and will include SDOH considerations in all our organizational efforts and policies. We will encourage partnerships across disciplines for practice, organizational leadership, education, research, and advocacy. We will work intentionally to build community and interdisciplinary partnerships. The underpinnings of unjust distribution of SDOH will guide SGIM’s work using ethical principles. We also encourage our general internist members to carry these considerations into their daily practice, their advocacy, their research portfolios, their organizations, and their teaching responsibilities and will develop tools to support them in this work.
